# CT-Guided Radiofrequency Thermal Ablation for the Treatment of Atypical, Early-Onset Osteoid Osteoma in Children Younger than 4 Years Old: Single-Institution Experience and Literature Review

**DOI:** 10.3390/diagnostics12112812

**Published:** 2022-11-16

**Authors:** Nicolas Papalexis, Giuliano Peta, Federico Ponti, Gianmarco Tuzzato, Marco Colangeli, Giancarlo Facchini, Paolo Spinnato

**Affiliations:** 1 Diagnostic and Interventional Radiology, IRCCS Istituto Ortopedico Rizzoli, 40136 Bologna, Italy; 2 Department of Orthopaedic Oncology, IRCCS Istituto Ortopedico Rizzoli, 40136 Bologna, Italy

**Keywords:** bone neoplasms, ablation techniques, interventional radiology, osteoid osteoma, pediatrics, image-guided biopsy

## Abstract

The aim of our study is to report our experience on CT-guided radiofrequency ablation (RFA) for osteoid osteoma (OO) in children under 4 years of age and to review the literature regarding this atypical, early onset of the disease. We retrospectively reviewed the clinical and radiological records of the patients treated with CT-guided RFA for OO at our institution (2006–2021), including those under 4 years of age. Data regarding technical success, clinical success, and biopsy diagnostic yield were collected. Moreover, we performed a literature review including previous articles on early-onset OO. We found only 12 patients that were under 4 years of age (12/842–1.4%) at the time of RFA treatment: 4 F and 8 M, mean age at the time of the treatment 35.3 months (range 22–46 months). The mean follow-up was 22.8 months (range 6–96 months). Technical success was achieved in all cases (12/12). In all patients (12/12), a complete remission of the pain symptoms was achieved at clinical follow-up controls. No recurrence of pain or complications were documented. The histopathological diagnosis was confirmed in 4 patients (4/12–33.3%). Moreover, we found another 9 articles in the literature with a main focus on early-onset OO (<4 years old), with a total of 12 patients included; 6 of those patients (6/12–50%) were treated with CT-guided RFA, with success reported 5 cases (5/6–83.3%). Our series of cases treated at a single institution, together with the existing data from the literature, confirms that CT-guided RFA is effective and safe for the treatment of osteoid osteoma, even in atypical, early onset in children under 4 years of age.

## 1. Introduction

Osteoid osteoma (OO) is a benign osteoblastic bone lesion first described by Jaffe in 1935 [1]. Osteoid osteoma represents the third most common benign bone tumor found in the population (10–12%) after osteochondromas and giant cell tumors of bone [2]. It usually affects patients between 5 and 30 years old although it can be found at any age. 

Typically, these tumors affect the long bone (i.e., femur and tibia), and the regions affected most frequently are the femoral neck and intertrochanteric region [3]. Osteoid osteomas are usually located within the cortical aspect of the bone (subcortical, intracortical, and intraperiosteal) [4]. Nevertheless, rarer locations such as medullary have been described [5]. Pain is the most common symptom: it is often referred to as a dull, intermittent pain that worsens over time. The pain is usually more intense during nighttime and greatly relieved using nonsteroidal anti-inflammatory drugs (NSAIDs). Other symptoms may include swelling, tenderness, or other symptoms specific to the location of the osteoma. In case of articular involvement, especially at the knee (distal femur or proximal tibia), the patient can complain of a reduction in the joint range of motion and an antalgic gait [6]. The imaging features of osteoid osteomas are peculiar. They have a characteristic radiolucent “nidus” less than 1.5 or 2 cm in diameter, surrounded by osteosclerotic reaction and bone oedema well-detectable on magnetic resonance imaging (MRI) scans. The “nidus” is usually detectable on computed tomography (CT) scans due to its small dimension, and it represents alone the neoplastic process [7,8]. In the last decades, minimally invasive interventional procedures have replaced open surgery for the treatment of OO. Currently, CT-guided radiofrequency thermal ablation (RFA) is the standard treatment technique for this condition [9,10]. Simultaneously, surgery is no longer indicated for the treatment for this conditions, with the exception of some locations. This tool has been demonstrated to be safe and effective in the treatment of osteoid osteoma, and it has the advantage of combining the treatment with CT-guided biopsy. This can be useful, particularly for atypical cases. CT-guidance was demonstrated to be safe and effective for interventional procedures in almost all skeletal sites, including the riskiest and most delicate ones, such as the craniovertebral junction (C0–C3) or the skull [11,12]. 

Other new interventional radiology techniques, such as MR-guided high-intensity focused ultrasound (MRgFUS), microwaves, or laser ablation, can be safely and effectively used for this aim as well [10]. Despite the large use in clinical practice of CT-guided RFA for OO treatment, there is a lack of data in regards to its application in atypical, early-onset osteoid osteoma, particularly in children younger than 4 years old. Indeed, the occurrence of this condition at this age is exceedingly rare, with only a few case reports in the literature [13]. Nonetheless, to the best of our knowledge, no literature is available focusing on the CT-guided treatments (or other minimally invasive procedures) for OO in children younger than 4 years old. The aim of our study was to report the experience of our referral center in the CT-guided RFA treatments of this condition with an atypical, early onset. Moreover, as a secondary aim, we performed a literature review of previous articles focused on this atypical, early onset in patients with OO.

## 2. Case Series Report from a Single Institution: Our Experience

We retrospectively reviewed all the clinical and radiological records of the patients treated with CT-guided RFA for osteoid osteoma at our Institution from January 2006 to December 2021 that were under 4 years of age at the time of the treatment. The diagnosis was determined from the patient history, radiological, and clinical examination findings (presence/absence of restlessness and crying at night). All patients had laboratory testing, including standard blood counts as well as coagulation and renal function checks. Parental or legal guardian refusal to agree, uncontrolled INR, and systemic or local illness were among the exclusion criteria. All procedures were performed by the same team of four interventional radiologists with at least 5 years of experience in skeletal interventional procedures (including PS and GF). All procedures performed in studies involving human participants were following the ethical standards of the institutional and/or national research committee and with the 1964 Helsinki Declaration and its later amendments.

### 2.1. Outcome Assessment

CT-guided RFA complications were documented according to the Society of Interventional Radiology classification [14]. Patients were clinically monitored for a minimum of 24 h after each procedure for evidence of acute complications (e.g., hematoma formation, neurologic injuries). The electronic medical records were reviewed for evidence of delayed complications within 30 days of the procedure.

Technical success was assessed with an intra-procedural CT scan and defined as the placement of the active tip of the needle in the center of the nidus of the osteoid osteoma. Clinical success was assessed at the follow-up clinical visits with reported complete remission of pain symptoms, disappearance of sleep awakenings, and discontinuation of NSAIDs intake. CT-guided biopsies were considered diagnostically successful if the histopathological analyses on the biopsies specimens permitted to confirm the final diagnosis. According to our institutional policy, after the last clinical follow-up, the patients have the possibility of contacting our referral center in order to report their clinical status or any recurrence in symptoms or to eventually have an additional clinical visit.

Imaging follow-up controls were not included in our institution guidelines for OO after CT-guided RFA. However, we checked our radiological archives to determine if imaging studies were available after treatments were checked by a radiologist with more than 10 years of experience in musculoskeletal radiology (PS) to assess for complications. 

### 2.2. CT-Guided Radiofrequency Ablation

CT-guidance was performed using 3 mm slice CT at 1.5 or 2 mm intervals, technical parameters 120 kV, milliamperage depending on the location of the osteomas (limbs, 150 mA; pelvis, 300 mA), and bone algorithm; for multiplanar reconstructions, a final CT data acquisition was performed from the proximal to the distal end of the lesions. CT scans were acquired with GE Lightspeed 4 slice (General Electrics, Boston, MA, USA) from 2006 to 2013 and with Brilliance CT 16 slice (Philips Medical Systems, Cleveland, OH, USA) equipment from 2013 to 2021. All procedures were performed under general anesthesia due to the young age of the patients. A pre-procedural CT scan of the lesion was acquired in order to choose the site of access and plan the percutaneous approach. A bone trocar (Bonopty Bone Biopsy System 14G, Apriomed, Uppsala, Sweden or Arrow OnControl, Telefex, Shavano Park, TX, USA) was introduced in the nidus of the osteoid osteoma, assessing the approach using consecutive CT images. Once in the center of the nidus, the bone biopsy needle was inserted coaxially in order to obtain a bioptic sample. The coaxial system was used to insert a 15 cm long, 17-gauge radiofrequency electrode with a 1/1.5 cm (depending on nidus dimensions and morphology) single active tip into the osteoid osteoma’s nidus. The trocar was then pulled out until it was beyond the anticipated ablation zone, and the ablation session was carried out at 90 °C for 6 min. After the ablation procedure, a post-procedural CT scan was performed to evaluate any immediate complications. For post-RFA pain treatment, patients were kept overnight, and pain was treated according to our institution’s post-operative acute pain service protocols for pediatric patients.

### 2.3. Patients Included in the Series (RFA for OO < 4 Years Old)

Among 842 patients treated with CT-guided RFA for osteoid osteoma in our institution (time period 2006–2021), only 12 were under 4 years of age (12/842–1.4%) at the time of the procedure.

Twelve patients included in our institution database were younger than 4 years old at the time of the CT-guided OO RFA treatment and were included in the study: four females and eight males, with a mean age at the time of treatments of 35.3 months (range 22–46 months). The mean patients’ age at the time of symptoms onset was 27.9 months (range 14–35 months). The mean interval from symptoms onset to treatment was 7.4 months. The mean size of the nidus was 9.6 ± 1.3 mm (range 6–12 mm). The most common skeletal locations were the femur (8/12–66.7%), followed by the tibia (3/12–25%) and the humerus (1/12–8.3%).

**Figure 1:**
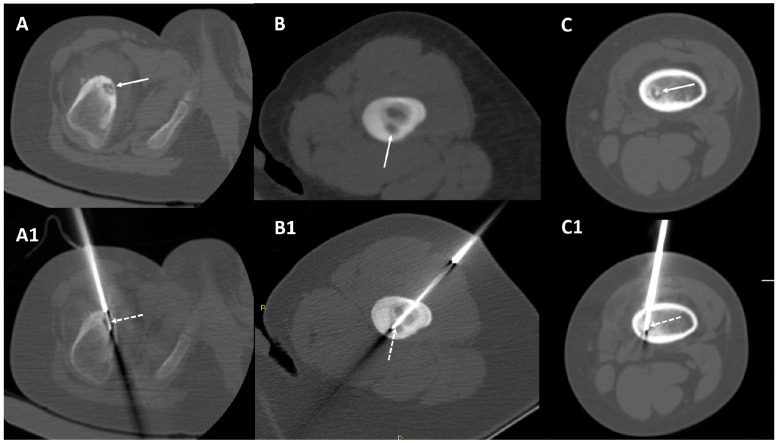
Three examples of technical success of the procedures; the tips were placed at the centers of the nidus (dotted arrows). Panel (**A**,**A1**)—a 46-month-old female with intracortical osteoid osteoma in the cortex of the right proximal femour (arrow). Panel (**B**,**B1**)—a 24-month-old male with cortical-endosteal osteoid osteoma located in the proximal left femour (arrow). Panel (**C**,**C1**)—a 33-month-old male with intramedullary osteoid osteoma located in the distal metaphysis of the left femour (arrow).

The 1.0 cm active tip was used in six patients (6/12–50%), while the 1.5 cm active tip was used in the remaining six subjects. All the cases were technically successful, with the active tip of the electrode being successfully placed in the center of the lesion (Figure 1).

At least one post-procedural clinical follow-up was performed in all patients (100%). Moreover, five patients (5/12–41.6%) had more than one follow-up visit. The mean follow-up was 22.8 months (range 6 to 96 months). None of the patients’ parents after the last clinical follow-up contacted our referral center for a recurrence of symptoms. 

In all patients (12/12–100%), the treatments resulted in clinical success, with complete remission of the pain symptoms achieved at clinical follow-up controls (*p* < 0.0001). All patients reported having stopped analgesic drug consumption at the first follow-up and a significant reduction of nocturnal awakenings. No recurrence of pain was documented in any of the patients. No complications were recorded after the procedures.

**Figure 2:**
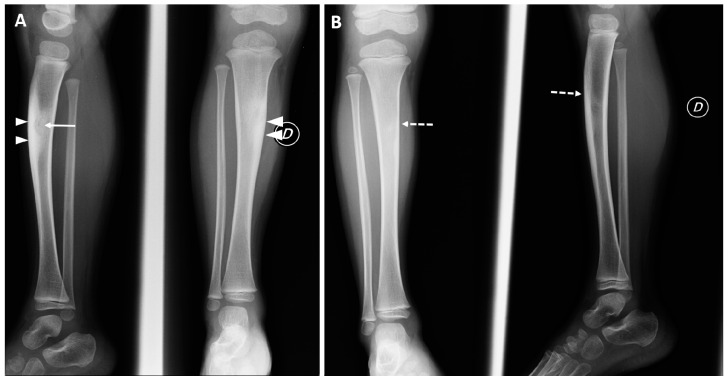
Conventional radiographs of a 35-month-old male (at the time of the treatment), performed after 2 months (Panel (**A**)) and 18-month follow-up (Panel (**B**)). Cortical thickening, mild tibial shape deformity (arrowheads), and the presence of the nidus (arrow) were still detectable at first control. Complete normalization of radiographic findings (dotted arrows) were assessed at the second follow-up control.

**Figure 3:**
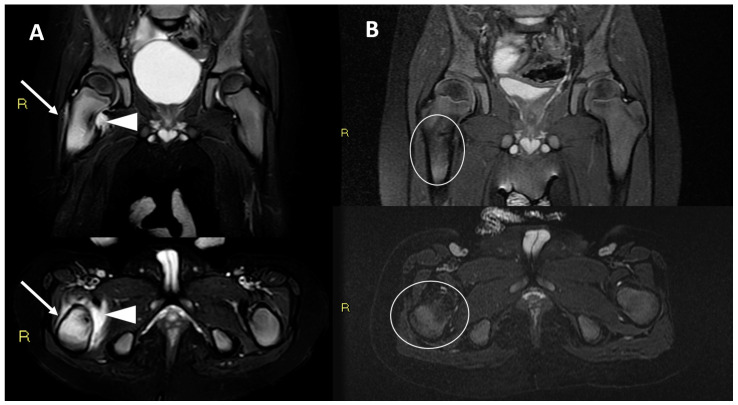
T2w fat saturated MRI images (coronal and axial) of a 27-month-old child male (at the time of the treatment), performed 2 months before (Panel (**A**)) and 6 months after radiofrequency ablation (Panel (**B**)). Before the treatment, wide bone oedema was detectable around the nidus in the right proximal femur (arrows); extra osseous soft-tissue oedema and fluid effusion were also detectable (arrowheads). A complete normalization of intraosseous and soft-tissue findings was achieved at 6-month MRI follow-up control (oval lines).

In the radiological hospital archive, we found imaging follow-up controls in three patients only (3/12–25%), two conventional radiographs, and one MRI, all with unremarkable findings (Figure 2 and Figure 3).

The main data of patients included and treatment outcomes are summarized in Table 1.

## 3. Literature Review

We performed a literature review (*PubMed* database) including the articles with a main focus on osteoid osteoma with early onset in children under 4 years of age to merge our experience with the relevant similar cases reported.

### 3.1. Literature Search Strategy

*MedLine* (via *PubMed*) database was searched up to 1 November 2022 using the string (“osteoid osteoma”) AND (“children” OR “infant” OR “toddler” OR “young”). Additionally, relevant keywords were used in different combinations for free-hand search, and the bibliography of selected articles was reviewed. Only clinical studies reporting OO in patients younger than 4 years of age were included in the final results.

### 3.2. Literature Search Results

In the literature, we found nine articles with a main focus on osteoid osteoma with onset before the 4 years of age, with a total of 12 patients included [13,15,16,17,18,19,20,21,22]. Six of those patients (6/12–50%) were treated with CT-guided RFA, with success reported in five cases (5/6–83.3%) and disease recurrence in only one (1/6–16.7%).

Moreover, some other research articles that were focused on osteoid osteoma in various age groups sporadically included in their series children under 4 years of age [23,24]. Moreover, their results are not detailed on this age group, and consequently, we cannot included them in our review analysis [23,24].

In Table 2, we report the main results from the articles found on *PubMed* with a main focus on osteoid osteoma in children younger than 4 years of age. 

## 4. Discussion

In our large series of 842 patients treated with CT-guided RFA for OO, only 1.4% of those (12 patients) were younger than 4 years old at the time of the procedure. The occurrence of OO with early onset in children younger than 4 years old is infrequent, with only some case reports described in the literature [13]. In this atypical and peculiar age group, OO could be difficult to diagnose, and a careful imaging evaluation (MRI, CT) is crucial to detect and treat this condition.

This is the largest series of atypical, early-onset OO in children younger than 4 years old, and the only series focused on minimally invasive treatment in this age group. Indeed, recent research focused on CT-guided RFA for OO in children included patients in the usually expected age range (5–18 years old) [25]. The results of our analyses are in line with the previous series focused on adults and/or children in regard to the safety and effectiveness of this treatment [26,27]. 

In our series, due to the few patients included, we did not observe disease recurrence. This eventuality varies mainly depending on lesion locations and is reported to account for 2% of cases for non-spinal OO and for 6–12% for spinal ones [25,26,27,28,29]. Nonetheless, it is well-known that some OO locations may not be safely treated with RFA, particularly due to the proximity to neural structures (e.g., particular spinal locations). Due to this, in these selected cases, surgery is still considered the first treatment option, and cases should be discussed at a multidisciplinary meeting by musculoskeletal interventional radiologists together with surgeons [27].

CT-guided RFA is currently the most used and efficacy-proven tool for OO treatment, and in light of recent research, other techniques can be safely used for this condition as well even if they have different advantages and technical peculiarities. Above all, MRgFUS is a very interesting tool, and due to the absence of ionizing radiation, it can be proposed as a valid alternative to CT-guided RFA, particularly in children and young adults [30].

The diagnosis of OO is mainly radiological and clinical, without the need of biopsy in most of typical cases. When a biopsy is performed, the histopatological confirmation is made usually in less than a half of patients. In our series, CT-guided biopsy performed in the same session of the RFA procedures resulted in a histopathological diagnosis of OO in four patients only (33.3%), similarly to the diagnostic yield already reported in other previous series [26]. 

In our series, only three patients (3/12) had a post-procedural imaging follow-up. Imaging follow-up after OO treatments is not indicated unless a relapse of symptoms occurs. After successful treatment, especially in young patients, the request for additional imaging studies depends on clinical/orthopaedic evaluation and should be related to regular growth check or assessment of suspected post-procedural complications.

Some pateints included in our series had a longer clinical follow-up than others. This was because they were referred to our center for other conditions such as trauma or other orthopedic issues even years after the RF ablation; therefore, clincal data on their previous RF ablation for OO was available, and we decided to include it in the study.

In conclusion, our article confirms that the occurrence of OO in children younger than 4 years old is possible even if it can be considered very rare (1.4% of all OO in our large population).

CT-guided RFA has been confirmed by our series and similar data in the literature to be safe and effective for the treatment of this condition even if applied in patients with atypical, early onset of the disease earlier than 4 years of age.

## Figures and Tables

**Table 1:** Main data of patients included and treatments outcomes.

Age at Onset of Symptoms (Months)	Age at Time of Treatment (Months)	Main Clinical/Anamnestic Data	Osteoid Osteoma Location	Nidus Max Diameter	Electrode Active Tip	Technical Success	Clinical Success	Histopathological Diagnosis	First Follow-Up	Last Follow-Up
30	38	Pain at night and limping	Femur proximal metaphysis, endosteal	9 mm	10 mm	Yes	Yes	Non-diagnostic	3 months after treatment: asymptomatic	15 months after treatment: asymptomatic
28	43	Pain at night	Tibia proximal metaphysis, endosteal	10 mm	15 mm	Yes	Yes	Non-diagnostic	1 month after treatment: asymptomatic	6 months after treatment: asymptomatic
27	33	Continuous pain worsened at night	Femur distal metaphysis, intramedullary	10 mm	15 mm	Yes	Yes	Non-diagnostic	6 months after treatment: asymptomatic	N/A
30	35	Continuous pain worsened at night	Tibia proximal metaphysis, cortical	9 mm	10 mm	Yes	Yes	Non-diagnostic	3 months after treatment: asymptomatic	18 months after treatment: asymptomatic
39	46	Pain at night and muscular hypotrophy	Femur proximal metaphysis, cortical-periosteal	10 mm	15 mm	Yes	Yes	Non-diagnostic	6 months after treatment: asymptomatic	N/A
22	37	Pain at night	Humerus diaphysis, endosteal	9 mm	10 mm	Yes	Yes	Osteoid osteoma	6 months after treatment: asymptomatic	N/A
14	22	Pain at night	Femur proximal metaphysis, endosteal	9 mm	10 mm	Yes	Yes	Osteoid osteoma	12 months after treatment: asymptomatic	N/A
20	28	Continuous pain and functional limitation	Femur proximal metaphysis, endosteal	7 mm	10 mm	Yes	Yes	Non-diagnostic	6 months after treatment: asymptomatic	8 years after treatment: asymptomatic
35	42	Continuous pain worsened at night	Tibia diaphysis, cortical-periosteal	12 mm	15 mm	Yes	Yes	Osteoid osteoma	4 months after treatment: asymptomatic	7 years after treatment: asymptomatic
34	39	Pain at night	Femur proximal metaphysis, cortical-periosteal	12 mm	15 mm	Yes	Yes	Non-diagnostic	4 months after treatment: asymptomatic	12 months after treatment: asymptomatic
32	34	Pain at night and restlessness	Femur diaphysis, cortical-endosteal	12 mm	15 mm	Yes	Yes	Osteoid osteoma	6 months after treatment: asymptomatic	N/A
24	27	Pain at night	Femur proximal metaphysis, cortical-periosteal	6 mm	10 mm	Yes	Yes	Non-diagnostic	4 months after treatment: asymptomatic	7 months after treatment: asymptomatic

**Table 2:** Main results of previous articles with a main focus on osteoid osteoma in children <4 years old.

First Author, Year, Reference Number	Study Design	Number of Patients	Patient Age	Diagnosis	Location	Clinical Findings	Treatment	Follow-Up	Recurrence
Bhat, 2003 Ref. [15]	Case report	1	27 months	OO	Femur	Limp on the medial distal thigh, which was swollen and tender	N/A	N/A	N/A
Ekstrom, 2006 Ref. [16]	Case report	1	7 months	OO	Femur	Restlessness and showed signs of pain at night but not during the day	CT-guided RFA	6 months	No
Virayavanich, 2010 Ref. [17]	Case report	1	7 months	OO	Femur	Decreased use of the right lower extremity due to pain	CT-guided RFA	3 months	Yes
Simon, 2013 Ref. [18]	Case report	1	14 months	OO	Femur	Pain, stiffness of the hip, and atrophy; coxa magna, limb discrepancy.	CT-guided RFA	8 years	No
Sahin, 2018 Ref. [19]	Case report	1	13 months	OO	Tibia	Restlessness for prior 6 months	CT-guided RFA	16 months	No
Laliotis, 2019 Ref. [20]	Case series	4	18 months–3 years	OO	2 femur, 1 tibia, 1 fibula	Pain, functional limitation, atrophy of the muscles (only femur)	Curettage for tibia and fibula, CT-guided RF for femur	12 months	No
Cotta, 2019 Ref. [21]	Case report	1	11 months	OO	Tibia	Limb asymmetry	N/A	N/A	N/A
Gupta, 2020 Ref. [22]	Case report	1	11 months	OO	Tibia	Inconsolable crying, swelling, and decreased use of left lower limb since the age of 8 months	Curettage	12 months	No
Hiramatsu, 2022 Ref. [13]	Case report	1	21 months	OO	Humerus	Affecter arm thinner than contralateral, restlessness for prior 5 months	Curettage	2 years	No

## Data Availability

The data presented in this study are available on request from the corresponding author.
